# First detection of the *kdr* mutation (L1014F) in the plague vector *Xenopsylla cheopis* (Siphonaptera: Pulicidae)

**DOI:** 10.1186/s13071-019-3775-2

**Published:** 2019-11-06

**Authors:** Nian Liu, Xiangyang Feng, Mei Li, Xinghui Qiu

**Affiliations:** 1 State Key Laboratory of Integrated Management of Pest Insects and Rodents, Institute of Zoology, Chinese Academy of Sciences, Beijing, 100101 China; 2 Guangxi Zhuang Autonomous Region Center for Diseases Control and Prevention, Nanning, 530028 China

**Keywords:** *Xenopsylla cheopis*, Voltage-gated sodium channel, Knockdown resistance, PCR-RFLP

## Abstract

**Background:**

The oriental rat flea, *Xenopsylla cheopis*, is the most efficient vector of the plague. Pyrethroid insecticides such as cypermethrin, cyhalothrin and deltamethrin have been often used to limit plague transmission *via* controlling the vector during outbreaks. However, this strategy is threatened by the development of insecticide resistance. Understanding the mechanisms underlying pyrethroid resistance is the prerequisite for successful flea control.

**Methods:**

Partial DNA sequences of *X. cheopis* voltage gated sodium channel (*VGSC*) gene were amplified from a total of 111 individuals, collected from a natural plague epidemic foci in Baise City, Guangxi Zhuang Autonomous Region of China. These DNA fragments were sequenced. The frequency and distribution of *kdr* mutations were assessed in four *X. cheopis* populations. The origin of *kdr* mutations was investigated by phylogenetic and network analysis.

**Results:**

The classical knockdown resistance (*kdr*) mutation (L1014F) was detected in four field populations at frequencies ranging between 0.021–0.241. The mutant homozygote was observed only in one of the four populations. Seven haplotypes were identified, with two of them carrying the resistance L1014F mutation. Phylogenetic tree and network analysis indicated that the L1014F allele was not singly originated. Based on polymerase chain reaction restriction fragment length polymorphism (PCR-RFLP) profiling, an easy-to-use and accurate molecular assay for screening individual fleas for the L1014F mutation was developed.

**Conclusions:**

To our knowledge, this work represents the first report of the L1014F mutation in the plague vector *X. cheopis*. The incidence of the L1014F allele highlights the need of further studies on the phenotypic effect of this mutation in this plague vector. Early detection and monitoring of insecticide resistance is suggested in order to make effective control strategies in case of plague outbreaks in this region.

## Background

The bubonic plague, caused by the bacterium *Yersinia pestis*, is a highly transmissible zoonotic disease. This disease is re-emerging with outbreaks occurring in many regions of the World [1]; 3248 cases were reported worldwide from 2010 to 2015, including 584 deaths [2]. The transmission of plague from rodents to humans is primarily caused by flea bites. The most efficient vector of the plague bacterium from rodents to humans is the flea species *Xenopsylla cheopis*; therefore, it has attracted increasing attention [3].

Plague remains an important health problem in China with 12 natural plague epidemic foci covering an area of 143.45 km^2^ in 19 provinces [4, 5]. Human plague once occurred in the region along the Tian-Sheng-Qiao Reservoir during 2000 to 2003 [5]. The Tian-Sheng-Qiao Reservoir was built in 1987, and water impoundment began from 1997 to 2000. This reservoir is located in the border region of three provinces (Guizhou, Guangxi and Yunnan), with a capacity of 10.3 billion m^3^. An epidemiological study documented that the outbreak of human bubonic plague was resulted from flea (*X. cheopis*) bites through rat-flea-human circulation [6]. The primary hosts of *X. cheopis* are *Rattus flavipectus* and *Rattus norvegicus* in this region [7, 8]. Since the plague outbreak, control of rats has been conducted twice a year (in spring and autumn) since 2002, usually followed by flea controls *via* spraying pyrethroids such as cypermethrin, cyhalothrin and deltamethrin on wall surface or ground surface [9].

Intensive use of insecticides can theoretically induce the development of resistance, ultimately leading to control failures. *Xenopsylla cheopis* populations were reported to be resistant to different classes of insecticides [3, 10, 11]. For example, 32 of 36 populations of *X. cheopis* were found to be resistant to deltamethrin in Madagascar [3]. However, the genetic basis underlying insecticide resistance in these populations remains unknown.

The voltage gated sodium channels (VGSC) are the primary target of pyrethroids in insects. VGSC mutation-mediated knockdown resistance (*kdr*) is the common and main cause of resistance to pyrethroids in insects [12–15]. Previous studies have documented more than 50 mutations or combinations of mutations associated with *kdr* in various arthropods [12], with the L1014F mutation in IIS6 being the first mutation that was detected and confirmed as a cause of *kdr* [13]. The conserved L1014F mutation was also characterized in the cat flea *Ctenocephalides felis* [14], and in the human flea *Pulex irritans* [15]. The present study aimed to investigate possible pyrethroid resistance-conferring genetic mutations in the voltage gated sodium channel, and to assess the geographical distribution of resistant genotypes in *X. cheopis* populations. Based on our results, a rapid diagnostic assay to detect the *kdr* mutation was established.

## Methods

### Flea samples

Fleas (*X. cheopis*) were collected in four different sites near the Tian-Sheng-Qiao Reservoir in Baise of Guangxi Province during 2017 and 2018, where plague outbreak occurred during 2000–2003 and pyrethroids have been extensively applied since then. Brief information about the sampling locations and dates is provided in Table 1. Flea samples were obtained according to the method of Nong et al. [9]. Briefly, live rats were trapped using randomly placed trap cages (100 cages/site), and brought to the laboratory; the trapped rat was placed into a sack filled with ether. Fleas were collected, classified morphologically, and stored in 100% alcohol at 4 °C.

**Table 1:** Brief information for *Xenopsylla cheopis* collection in Baise of Guangxi, China

Site	Location	Coordinate	Date
1	Yongle, Youjiang County	106°37′12″E, 23°59′24″N	October 2017
2	Wangdian, Youjiang County	106°21′4″E, 24°11′24″N	October 2017
3	Gebu, Longlin County	105°2′24″E, 24°47′24″N	June 2018
4	Bada, Xilin County	105°6′3″E, 24°29′24″N	April 2018

### Extraction of genomic DNA and amplification of *XC-VGSC* gene fragments

Genomic DNA of individual fleas was isolated by the method of Rinkevich et al. [16]. Primers (Xchvgsc-F: 5′-GTG CCT TGG GTA ATC TAA CGT-3′ and Xchvgsc-R: 5′-CGA CAA GAG CAA CGC CAA G-3′) were designed based on the partial DNA sequence of *X. cheopis* para-type voltage-gated sodium channel-like gene (GenBank: JQ894869.1), and used to amplify gene fragments (~ 440 bp) containing codon 1014 (*Musca domestica* numbering) within the domain II region of insect *para* sodium channel. The reaction mixture consisted of 12.5 μl of 2× SuperHiFi PCR Master Mix (DAKEWE Biotech, Shenzhen, China), each primer 5 μM, 75–150 ng DNA template, and ddH_2_O up to 25 μl. Reactions were programmed as 95 °C for 3 min, 42 cycles of 94 °C for 25 s, 55 °C for 25 s, 68 °C for 30 s, and extension of 68 °C for 10 min. PCR products were gel-purified and directly sequenced using primer Xchvssc-F (TSINGKE, Beijing, China).

### Sequence analysis

DNA sequencing data were checked manually. All the confirmed sequences were aligned with MUSCLE 3.8 [17], and nucleotide polymorphisms (SNPs) were documented. Hardy-Weinberg equilibrium (HWE) probability test was conducted using the online software GENEPOP v.4.2 [18]. The *VGSC* haplotypes were identified by directly reading from homozygotes, or by splitting one from the other from heterozygotes carrying one-site variations. Clone sequencing was conducted for four samples of 1014L/F heterozygotes with multiple-site variations. For clone sequencing, purified PCR products were ligated with the pEasy-T1 vector and transformed into competent cells of the *Escherichia coli* (DH5a). MEGA 7 [19] and Network v.4.6 [20] were used to analyze the evolutionary origin of the identified haplotypes.

## Results

### Detection of *kdr* mutations

**Fig. 1:**
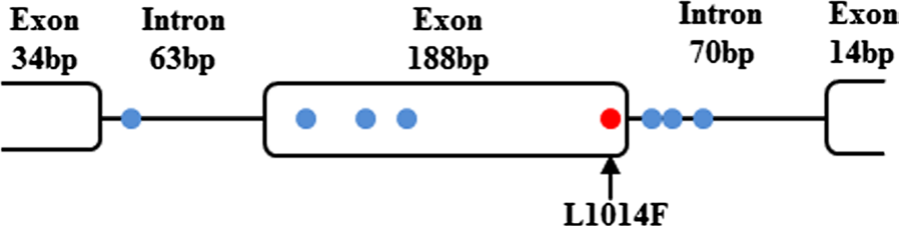
Schematic representation of the region of *Xenopsylla cheopis* *VGSC* gene analyzed in this study. Dots indicate the polymorphic sites in the obtained sequences. The red dot represents the site leading to L1014F mutation

Sequencing of each amplicon resulted in a clear 369-bp sequence, encompassing two full short introns, a full exon and two partial exons (Fig. 1). Eight nucleotide polymorphisms in the 369-bp fragments were observed from a total of 111 individuals. No nucleotide variation was observed in the first and last partial exons. One non-synonymous mutation, leading to a deduced amino acid substitution of L (CTT) to F (TTT) at the position corresponding to the amino acid residue 1014 of VGSC of the housefly was detected in the second exon, while other three nucleotide polymorphisms in this exon were synonymous. Four polymorphic sites were found in the two introns in total, without length polymorphism being observed (Fig. 1).

**Fig. 2:**
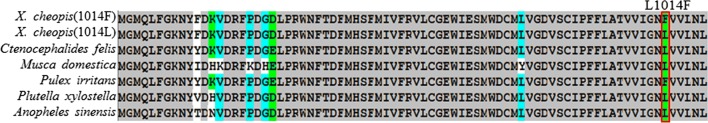
Multiple alignments of the partial deduced amino acid sequences of the *VGSC* gene from *Xenopsylla cheopis* (1014F and 1014L) with corresponding sequences from other species, including *Ctenocephalides felis* (CAG30671.1), *Musca domestica* (CAA65448.1), *Pulex irritans* (ANY39518.1), *Plutella xylostella* (ADH16760.1) and *Anopheles sinensi*s (ABC60026.1)

The putative partial amino acid sequences (79 amino acids) of *X. cheopis* VGSC (1014L and 1014F) showed high identities with VGSCs of species of the Pulicidae such as the cat flea *Ctenocephalides felis* (CAG30671.1, 97.44%) and the human flea *Pulex irritans* (ANY39518.1, 96.15%). High similarity of VGSC was also observed between *X. cheopis* with the dipteran *Anopheles sinensis* (ABC60026.1, 97.44%), and the lepidopteran *Plutella xylostella* (ADH16760.1, 97.44%) (Fig. 2).

### Distribution and frequency of *kdr* alleles

**Fig. 3:**
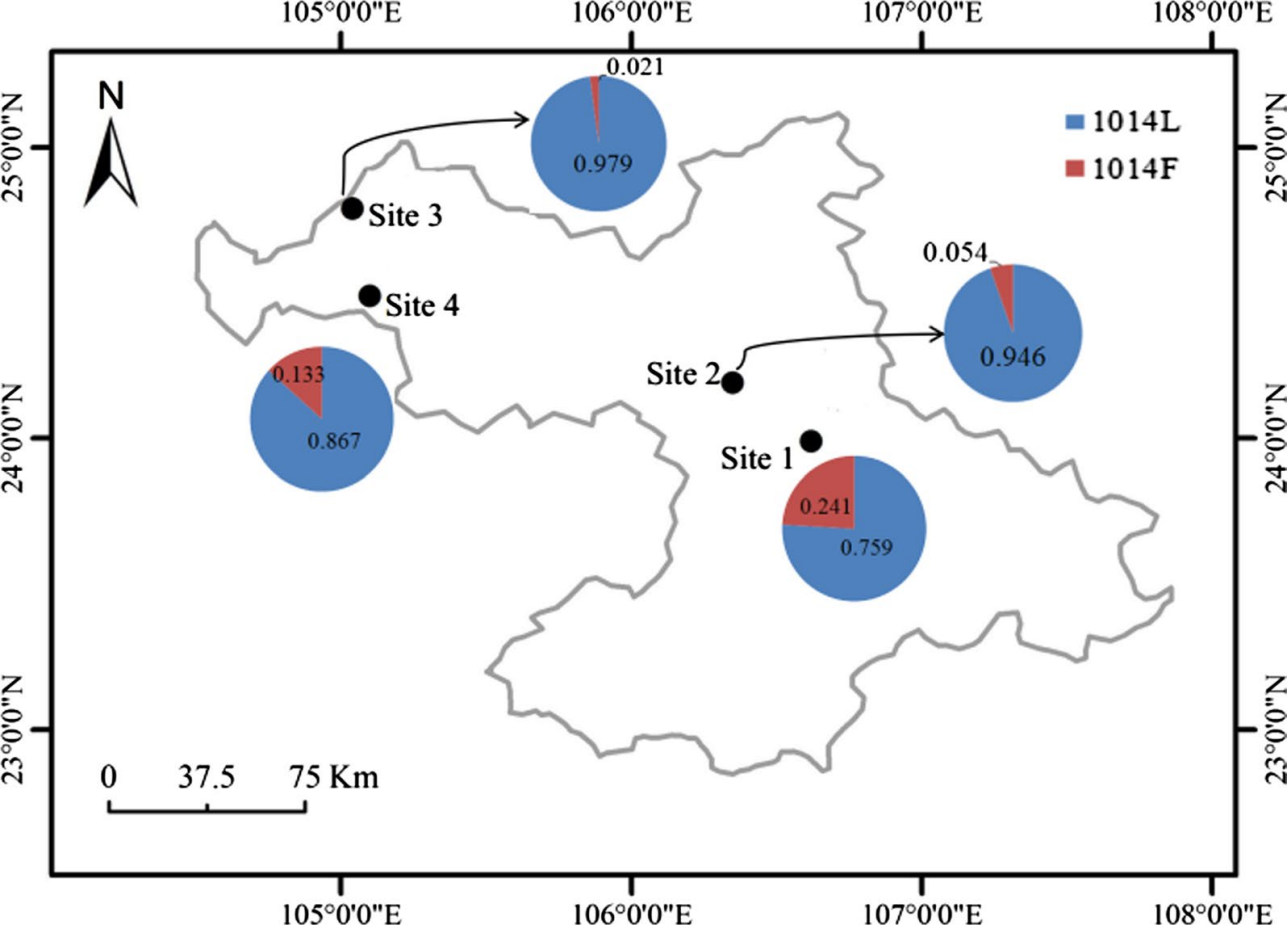
Distribution and frequency of *kdr* alleles in *Xenopsylla cheopis* populations. 1014L and 1014F stand for the susceptible allele and resistant allele, respectively

Focusing on codon 1014, the three possible genotypes of the *XC-VGSC* gene were observed, and all genotypes were found to agree with Hardy-Weinberg equilibrium (Table 2). The resistant 1014F allele was detected in all of the four populations, with the highest frequency of 0.241 being in Yongle, Youjiang County (YLYJ, Site 1) (Fig. 3). The *kdr*-resistant homozygote was rare and detected only in YLYJ at a low frequency (0.034). The wild-type homozygote was present at high frequencies (0.552–0.958), while the frequencies of heterozygotes varied from 0.042 to 0.414.

**Table 2:** Frequency of *Vgsc* genotypes in the four *Xenopsylla cheopis* populations from Baise of Guangxi, China

Site	*n*	Frequency			HWE probability test	
		LL	LF	FF	*P*-value	SE
1	29	0.552	0.414	0.034	1.0000	0.0000
2	28	0.857	0.143		1.0000	0.0000
3	24	0.958	0.042			
4	30	0.733	0.267		1.0000	0.0000

*Abbreviations*: HWE, Hardy-Weinberg equilibrium; SE, standard error

### Genealogical analysis of *XC-VGSC* haplotypes

**Fig. 4:**
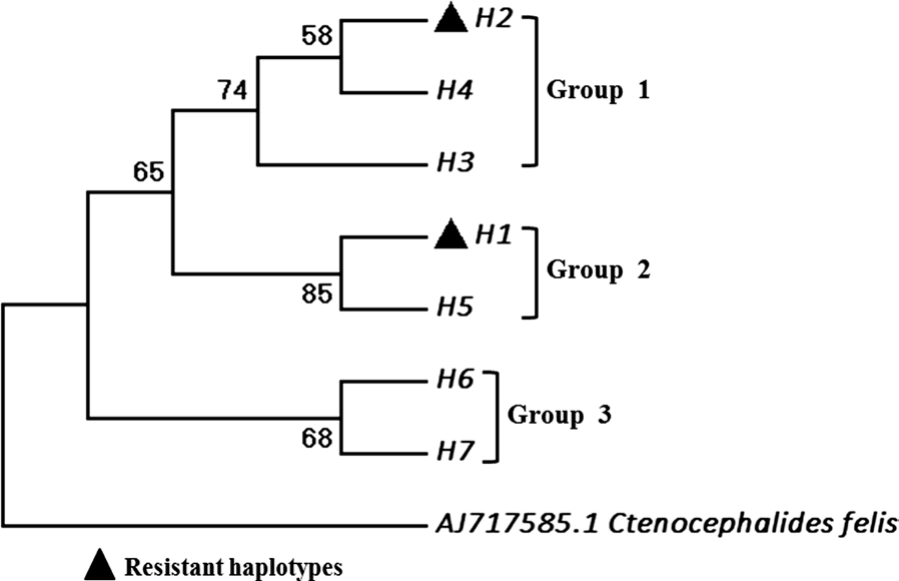
Phylogenetic tree generated using maximum likelihood method. The analysis involved the seven haplotypes identified in this study, and the corresponding region of the cat flea *VGSC* gene (AJ717585.1) as outgroup. Bootstrap test confidence values (%) from 1000 replicates are given on tree nodes. The resistant haplotypes are indicated by a filled triangle

**Fig. 5:**
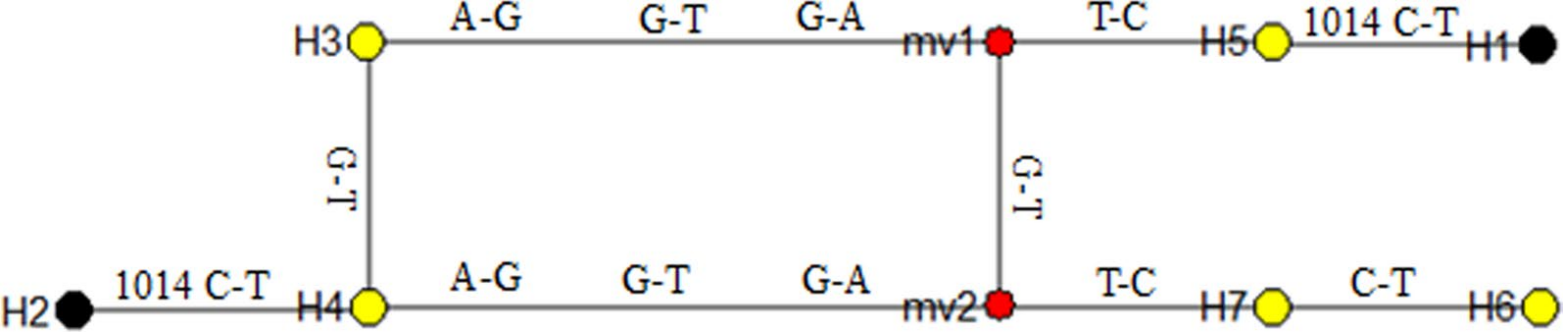
Genealogical relationship among the seven *Xenopsylla cheopis* VGSC haplotypes. The base variation between two haplotypes is listed above the line connecting the haplotypes. The resistant haplotype is indicated by a black dot and the susceptible haplotype is marked by a yellow dot. The median vectors (mv1 and mv2, red dot) are hypothesised sequences required to connect existing sequences within the network with maximum parsimony

Seven haplotypes (MN103328-MN103334) were identified in this study, with two of them (H1 and H2) carrying the L1014F mutation (Table 3). The DNA sequences of these haplotypes were used for reconstruction of a phylogenetic tree and for network analysis. The phylogenetic tree revealed that the seven haplotypes are clustered into three groups (Fig. 4). Notably, the two 1014F-carrying haplotypes (H1 and H2) was grouped into different branches (Group 1 and Group 2, respectively), and their nucleotide sequences differed in both introns and exon (Table 3). The network analysis indicated that H1 and H2 were derived from H5 and H4, respectively, through only one mutational step (Fig. 5). Sequence analysis showed that H1 and H5 had identical introns, while H2 shared the same introns with H4 (Table 3).

**Table 3:** Haplotypes of the *XC-VGSC* gene

Haplotype name	Nucleotide at the polymorphic sites	Amino acid at residue 1014	GenBank ID
H1	TCCTTATG	F	MN103328
H2	GTCTTGGA	F	MN103329
H3	TTCTCGGA	L	MN103330
H4	GTCTCGGA	L	MN103331
H5	TCCTCATG	L	MN103332
H6	GTTCCATG	L	MN103333
H7	GTCCCATG	L	MN103334

*Note*: the polymorphic sites in introns are underlined

### Establishment of PCR-RFLP assay for genotyping *XC-VGSC* 1014

**Fig. 6:**
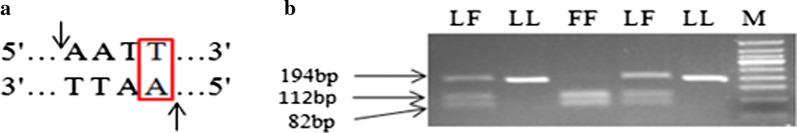
PCR-RFLP assay for *XC-VGSC* 1014 genotyping. a The digestion site of enzyme MluCI. The first nucleotide of codon 1014 of the 1014F allele is boxed. **b** Electrophoresis detection of restriction endonuclease digestion product. Lane M: DNA marker; L: 1014L; F: 1014F

The SNPs at codon 1014 of *XC-VGSC* generate a MluCI digestion site (Fig. 6a), allowing us to establish a PCR-RFLP assay to genotype *XC-VGSC* 1014. Two primers (Xchvgsc-SF: 5′-GTG GAT CGA GTC TAT GTG GGA-3′, which was designed based on a highly conserved region of DNA sequences, and Xchvgsc-R: 5′-CGA CAA GAG CAA CGC CAA G-3′) were used for PCR. The PCR mixture (25 μl) contained 2× EZ4SuperHiFi mix 12.5 μl; each primer 5 μM, 75–150 ng DNA template, and ddH_2_O. PCR conditions were as follows: 95 °C for 3 min, followed by 42 cycles of 94 °C for 25 s; 55 °C for 25 s and 68 °C for 30 s, and a final extension at 68 °C for 10 min. The digestion reactions contained 0.5 U of MluCI (New England Biolabs, Beijing, China), 2 μl of cutsmart buffer, 8 μl of PCR product, and ddH_2_O up to 20 μl. The digestion mixture was incubated at 37 °C for 3 h, and the digested products were run on 3% gel and visualized under UV. The genotypes were identified based on the presence or absence of cleaved bands. For 1014L alleles, there is no restriction site for MluCI, only a 194-bp band was present on the gel. MluCI was able to cut 1014F allele, giving two cleaved fragments of 112 bp and 82 bp. The RFLP profiles for the three possible genotypes are shown in Fig. 6b.

Direct sequencing of the PCR products was performed to validate the PCR-RFLP genotyping results. PCR-RFLP gave unambiguous scoring of all genotypes that were 100% in agreement with direct sequencing in a total of sixty individuals (10 from Sites 2–4, respectively, and 30 from Site 1).

## Discussion

Pyrethroids are widely and often used to control disease vectors. Following the continuous use of various pyrethroids, resistance has been documented in many insects [12]. A major mechanism of pyrethroid resistance, which is caused by point mutations in the voltage gated sodium channel, has been well recognized. The common L1014F mutation, originally found in *Musca domestica* and referred to as *kdr* mutation [13], has been detected in a wide variety of insect species [12], including the cat flea (*C. felis*) [14] and human flea (*P. irritans*) [15]. In this survey, the presence of the L1014F mutation was observed in all the four tested *X. cheopis* populations collected in Baise of Guangxi Province. To our knowledge, this represents the first detection of the classical *kdr* mutation (L1014F) in this plague vector.

The levels of resistance conferred by the L1014F mutation have been documented variable, ranging from 12 (fenpropathrin) to 260 (etofenprox) in the house fly [21]. The overall low frequency of the L1014F allele indicates that the *kdr* mechanism seems unlikely to cause failure of oriental rat flea control in this region. However, as a limitation of this study, the lack of susceptibility information for the samples tested in this study makes it risky to predict the status of insecticide resistance in these oriental rat flea populations. Additionally, it is unknown whether other factors such as increased detoxification of insecticides contribute to resistance. Therefore, further investigations are required to clarify this issue. The relatively high frequency (0.241) of the L1014F mutation (Fig. 3) and the occurrence of resistant homozygote (1014FF) in the YLYJ population (Table 2) calls for close monitoring and surveillance of pyrethroid susceptibility in this area.

Two different haplotypes that carry the L1014F mutation (H1 and H2) were identified (Table 3). Among the eight polymorphic sites, five were different between H1 and H2. Further phylogenetic and network analysis strongly suggest that H1 and H2 have independent origins (Figs. 4, 5). The two resistant haplotypes (H1 and H2) could be derived from wild H5 and H4 with one mutational step respectively. Multiple origins of insecticide resistance conferring point mutations in the voltage-gated sodium channel gene have also been characterized in several other insect species [17, 22–25].

Early detection and monitoring of insecticide resistance in a vector population may provide evidence-based implications for making vector intervention strategies. In this context, we have developed a simple PCR-RFLP assay that can be used to accurately genotype individual fleas for the resistance-conferring L1014F mutation (Fig. 6b).

## Conclusions

The classical *kdr* mutation (L1014F) was detected in *X. cheopis* field populations collected from Guangxi Province of China at frequencies of 0.021–0.241. Two different haplotypes carrying the L1014F mutation were identified. Phylogenetic tree and network analysis suggested that the L1014F allele was not singly originated. A simple, rapid and accurate molecular assay for screening individual fleas for the L1014F mutation was developed.

## Data Availability

Data supporting the conclusions of this article are provided within the article. The newly generated sequences were submitted to the GenBank database under the accession numbers MN103328-MN103334.

## Abbreviations

*Kdr*: knockdown resistance
PCR: polymerase chain reaction
RFLP: restriction fragment length polymorphism
VGSC: voltage gated sodium channel
